# Synovial Tissue Inflammation Mediated by Autoimmune T Cells

**DOI:** 10.3389/fimmu.2019.01989

**Published:** 2019-08-21

**Authors:** Yusuke Takeuchi, Keiji Hirota, Shimon Sakaguchi

**Affiliations:** ^1^Laboratory of Integrative Biological Science, Institute for Frontier Life and Medical Sciences, Kyoto University, Kyoto, Japan; ^2^Department of Rheumatology and Clinical Immunology, Graduate School of Medicine, Kyoto University, Kyoto, Japan; ^3^Laboratory of Experimental Immunology, Immunology Frontier Research Center, Osaka University, Osaka, Japan; ^4^Laboratory of Experimental Immunology, Institute for Frontier Life and Medical Sciences, Kyoto University, Kyoto, Japan

**Keywords:** autoimmune arthritis, Th17, treg cells, rheumatoid arthritis, synovial inflammation, proinflammatory cytokine

## Abstract

In rheumatoid arthritis (RA), various hematopoietic and non-hematopoietic cells present in the synovial tissue secrete numerous inflammatory mediators including pro-inflammatory cytokines critical for the induction of chronic joint inflammation and bone destruction. Fibroblast-like synoviocytes (FLSs) in the non-hematopoietic cell compartment are key inflammatory cells activated in inflamed joints and driving the disease; yet how synovial tissue inflammation is modulated by autoimmune T cells is not fully understood. In this review, mainly based on recent findings with a mouse model of spontaneous autoimmune arthritis, we discuss the mechanism of Th17-mediated synovial tissue inflammation; that is, what environmental stimuli and arthritogenic self-antigens trigger arthritis, how arthritogenic T cells initiate joint inflammation by stimulating FLSs, and how the cellular sources of GM-CSF from lymphoid and tissue stromal cells in the synovium contribute to the development of arthritis. We also highlight possible plasticity of Th17 cells toward pathogenic GM-CSF producers, and the functional instability of regulatory T cells under inflammatory conditions in RA joints.

## Introduction

Rheumatoid arthritis (RA), which afflicts 1% of the population worldwide, is a systemic autoimmune disease characterized by chronic inflammation of the synovial tissue and joint destruction (1). There is accumulating evidence that various types of immune cells (e.g., T cells, B cells, macrophages, and neutrophils) with pro-inflammatory or anti-inflammatory potentials are recruited into RA joints and play key roles for RA development and progress (1). Although the molecular and cellular mechanisms of RA pathogenesis are still controversial, it is now generally accepted that CD4^+^ T helper (Th) cells play a crucial role in disease manifestation of RA as indicated by abundant infiltration of Th cells in RA joints, human leukocyte antigen (HLA)-DRB1 identified as the strongest disease risk gene for RA, and high efficacy of CTLA4-Ig treatment in RA patients (1–3). On the other hand, it is evident that macrophage- and fibroblast-like synoviocye (FLS)-derived inflammatory cytokines such as TNFα, IL-1, and IL-6 are abundant in RA synovial fluid and involved in joint inflammation and destruction as well, illustrated by high effectiveness of anti-cytokine therapies targeting TNFα and IL-6 in controlling disease activities (4, 5). However, how Th cells interact with macrophages and FLSs or even other immune cells to orchestrate chronic synovial inflammation is poorly understood. In this review, we discuss how autoimmune Th17 cells instigate FLSs and innate lymphoid cells (ILCs) in the synovial tissue to initiate and maintain autoimmune arthritis in SKG mice (6), an animal model of RA, how arthritogenic Th17 cells differentiate toward pathogenic GM-CSF producing cells in RA, and how regulatory T (Treg) cells become functionally unstable in inflamed RA joints.

## Th Cell-Dependent Autoimmune Arthritis in SKG Mice

There are several mouse models of autoimmune arthritis that have made significant contributions to our understanding of the pathogenetic mechanisms of RA. For example, collagen-induced arthritis (CIA) and K/BxN mice are highly dependent on the generation of autoantibody against type II collagen and glucose-6-phosphate isomerase, respectively, as the serum alone, without cellular components, from arthritic mice is able to transfer the disease into recipient mice (7–9). By contrast, self-reactive Th cells, but not autoantibody, mediate joint inflammation in SKG mice, making the mice a suitable model to understand how autoimmune T cells initiate and mediate synovial inflammation.

The SKG strain of mice on the BALB/c background spontaneously develops Th cell-mediated autoimmune arthritis as a consequence of a recessive point mutation in the gene encoding ζ-associated protein-70 (Zap-70), a key TCR-proximal signaling molecule (6, 10). This mutation impairs positive and negative selection of T cells in the thymus, leading to the production of self-reactive CD4^+^ T cells including arthritogenic Th cells and reduced thymic production of Treg cells. The resulting autoimmune arthritis resembles human RA in immunopathology, for example, in abundant infiltration of Th cells into arthritic joints and active formation of autoantibodies such as rheumatoid factor and anti-citrullinated peptide antibodies (6, 10, 11). Spontaneous arthritis in SKG mice occurs in a conventional environment, but not under the specific-pathogen-free condition (10, 12). Yet arthritis can be triggered by activating innate immunity via Toll-like receptors, the Dectin pathway, or complement pathways, for example, by injection of zymosan, a crude fungal extract containing β-glucans. It is also induced by allowing homeostatic proliferation of arthritogenic CD4^+^ T cells in lymphopenic mice such as *Rag2*^−/−^ mice (12, 13). Recently, we have identified 60S ribosomal protein L23a (Rpl23a) as an arthritogenic self-antigen in SKG mice by single-cell TCR repertoire analysis of effector Th cells isolated from inflamed joints and then screening arthritogenic potentials of isolated TCRs in retrogenic mice expressing a single TCR. Moreover, autoantibody against RPL23A was specifically detected in the sera from RA patients, which further illustrates the similarity in the molecular basis of the joint inflammation between SKG arthritis and human RA (14). Additionally, SKG mice were reported to be a disease model of spondylarthritis since they develop enthesitis, dactylitis, unilateral uveitis, sacroiliac joint arthritis, and vertebral inflammation together with peripheral joint arthritis, which are the clinical features of spondylarthritis (15, 16).

Among Th cell subsets, we showed that IL-17-producing T helper (Th17) cells played a key role in the development of arthritis in SKG mice (17). Cell transfer of CD4^+^ T cells from *Il17a*^−/−^ SKG mice into T cell-deficient mice completely failed to induce arthritis. *Il6*^−/−^ SKG mice, which were impaired in T cell differentiation into Th17 cells, hardly developed arthritis. Although both Th1 and Th17 cells were detected in inflamed joints of SKG mice, IFN-γ-deficiency did not ameliorate, but rather exacerbated SKG arthritis because IFN-γ-deficiency augmented the differentiation and pathogenic function of Th17 cells. Similar findings in IFN-γ-deficient or its receptor deficient mice were also made in other Th17-mediated disease models such as CIA and experimental autoimmune encephalomyelitis (EAE), a well-established murine model of multiple sclerosis (18, 19). This anti-inflammatory effect of IFN-γ can be explained, at least in part, by decreased expression of indoleamine 2,3 dioxygenase (IDO), a catabolic enzyme responsible for kynurenine product from tryptophan. IDO is expressed in various types of cells including macrophages and dendritic cells (DCs) and is induced during inflammation by IFN-γ or other inflammatory cytokines. Increased levels of IFN-γ-mediated IDO expression in DCs in inflamed tissues play a key role in immune regulation in part by deprivation of local tryptophan, followed by activation of the general control non-repressed 2 kinase pathway in responding to T cells, leading to inhibition of activation and proliferation of local T cells including pathogenic Th17 cells. By contrast, IFN-γ deficiency downregulates IDO expression, which subsequently allows pathogenic Th17 cells to further expand in the sites of inflammation and augment autoimmune pathologies (20–23). These findings taken together support SKG mice as a suitable animal model to address how autoimmune T cells orchestrate chronic joint inflammation (17).

## Initiation of Arthritis by Interaction Between Arthritogenic Th17 Cells and Fibroblast-Like Synoviocytes

The initiation of arthritis in SKG mice requires self-reactive (arthritogenic) CD4^+^ T cells to differentiate into effector Th17 cells and migrate into the joints. As the mechanism of this Th17 differentiation of self-reactive CD4^+^ T cells, they first become activated in the periphery by strong recognition of class II MHC/self-peptide complexes expressed by antigen-presenting cells (APCs), and reciprocally stimulate APCs to upregulate CD80/CD86 through CD40/CD40L interaction, further enhancing the activation and proliferation of self-reactive CD4^+^ T cells. The APCs thus stimulated secrete IL-6, IL-1, and IL-23, which, together with local tissue-derived TGFβ, drive the differentiation of naïve CD4^+^ T cells into arthritogenic Th17 cells. The migration of the arthritogenic Th17 cells into the synovial tissue occurs in a chemokine-dependent manner, in particular via the CCR6-CCL20 axis. That is, Th17 cells predominantly express CCR6, while activated FLSs secrete CCL20, the ligand of CCR6, so that FLSs preferentially recruit Th17 cells into the synovial tissue. In fact, anti-CCR6 blocking monoclonal antibody significantly decreased the severity of arthritis in SKG mice by inhibiting infiltration of Th17 cells into the joints (24). Moreover, FLSs upregulate CCL20 expression in response to IL-17, IL-1, or TNFα, whereas IFN-γ or IL-4 inhibits the expression (24). These observations illustrate an important feed-forward interaction between Th17 cells and FLSs in the initiation and augmentation of joint inflammation: once arthritogenic Th17 cells are activated and recruited into the joints to initiate inflammation, FLSs further increase CCL20 in response to IL-17 and other pro-inflammatory cytokines, and accelerate the recruitment of Th17 cells to initiate and augment arthritis (24). Activated FLSs interact not only with Th17 cells, but also with other immune cells to establish chronic synovial inflammation, for example, by enhancing secretion of chemokines such as Cxcl1 and Cxcl5 to recruit neutrophils into the joints (25). Interestingly, similar to these findings in SKG mice, Th17 cells and synoviocytes in RA patients express CCR6 and CCL20, respectively, with a significant correlation between the level of IL-17 and CCL20 in RA joints (24). Furthermore, recent GWAS studies have identified the CCR6 gene as a disease susceptibility gene locus of RA. These observations collectively indicate the interaction between Th17 cells and FLSs as a key mechanism for the development of synovial tissue inflammation (26).

## GM-CSF, a Key Mediator That Maintains Chronic Joint Inflammation

Granulocyte macrophage colony-stimulating factor (GM-CSF) from various sources in inflamed synovial tissues amplifies chronic synovial inflammation. We have recently demonstrated that GM-CSF from lymphoid and tissue-resident cells in the joint is a key component for initiating and maintaining autoimmune arthritis (25). GM-CSF has recently been highlighted as a potent pro-inflammatory cytokine that activates monocytes and macrophages, and is produced by various types of cells, for example, fibroblasts, endothelial cells and T cells in response to inflammatory stimuli (27). GM-CSF can be targeted for the treatment of RA because it is abundant in RA synovium in association with strong activation of synovial macrophages, and active production of IL-6 and TNFα in RA joints (28, 29). In addition, *Csf2*^−/−^ SKG mice completely failed to develop arthritis regardless of the presence of effector Th17 cells in the periphery, indicating an indispensable role of GM-CSF in SKG arthritis (25). GM-CSF was partly produced by effector Th17 cells in inflamed joints; however, T-cell-derived GM-CSF—although it augmented arthritis—was dispensable for the induction of arthritis, while non-T cell-derived GM-CSF was indispensable. By generating mixed bone marrow chimeras, we have identified radio-resistant stromal cells including FLSs and synovial-resident ILCs as the crucial source of GM-CSF. Indeed, the severity of arthritis in the bone marrow chimeras was significantly reduced when GM-CSF production was specifically inhibited or lost either in radio-resistant stromal cells or ILCs. Re-stimulation with IL-17 induced GM-CSF production from FLSs *in vitro*. Moreover, when Th cells from wild-type SKG mice, but not *Il17a*^−/−^ SKG mice, were adoptively transferred into *Rag2*^−/−^ mice, synoviocytes significantly increased the expression of *Csf2* together with *Il6* and *Ccl20*. These observations when taken together imply that IL-17 from effector Th17 cells migrating into the synovial tissue stimulates FLSs to produce GM-CSF. A small number of ILCs are present in healthy joints of SKG mice as well as other healthy mouse strains; among them, GM-CSF-producing ILCs have substantially expanded in inflamed joints. Interestingly, a large proportion of these GM-CSF-producing synovial ILCs possessed the features of the group 2 ILCs (ILC2s). For example, they predominantly express GATA3 and/or IL-13, known as the master transcription factor and a signature cytokine, respectively, of ILC2s, and upregulated *in vitro* production of GM-CSF as well as IL-5 and IL-13 in response to a combination of IL-2 and IL-33 stimulation. In addition, these synovial ILCs highly express Toll-like receptor 9 (Tlr9) and synergistically increase GM-CSF production when stimulated by a combination of IL-33 and CpG DNA, a ligand of TLR9, but not by CpG DNA alone. It is thus likely that synovial ILCs may respond to signals from necrotic cells in inflamed joints, such as alarmins and damage-associated molecular patterns (DAMPs) including IL-33 and mitochondrial DNA. These findings taken together indicate that, unlike FLSs, synovial ILCs become activated, expand and increase GM-CSF production by sensing environmental signals such as IL-2, IL-33, and self-DNA, which could be derived from arthritogenic Th17 cells and damaged cells in inflamed joints (25).

**Figure 1:**
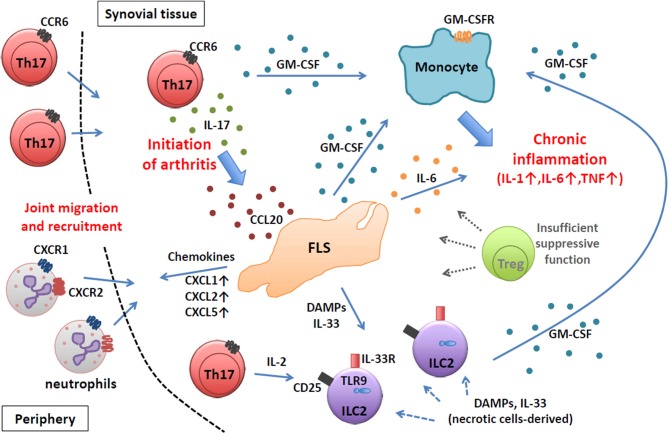
Chronic joint inflammation mediated by FLSs and inflammatory immune cells in SKG arthritis. Arthritogenic Th17 cells migrate into synovial tissue and initiate joint inflammation by stimulating FLSs via IL-17. Activated FLSs secrete various inflammatory mediators to recruit and expand various inflammatory immune cells. At a chronic stage, FLSs, ILC2s, and Th17 cells produce GM-CSF to activate inflammatory monocytes to facilitate chronic joint inflammation.

Thus, in autoimmune arthritis in SKG mice, which is fully dependent on Th17 cells, the key primary event in the initiation of arthritis is the migration of Th17 cells into the synovial tissue to instigate FLSs by IL-17 and to modulate their inflammatory profiles. Following the initiation of arthritis, arthritogenic Th17 cells together with FLSs and synovial ILCs orchestrate an inflammatory cascade to amplify chronic joint inflammation by GM-CSF from various sources subjected to different immunological stimuli, resulting in the activation of synovial macrophages and their abundant production of pro-inflammatory cytokines such as IL-1, IL-6, and TNFα (Figure 1).

## Th Cells in Rheumatoid Arthritis

Before the discovery of Th17 cells, RA was believed to be a Th1-mediated autoimmune disease based on the classical Th1/Th2 paradigm in autoimmunity. Indeed, IFN-γ^+^ Th cells are abundant among CD4^+^ T cells in RA synovial fluid (SF) (30, 31). However, the pathogenic roles of IFN-γ in RA were unclear because the intervention of IFN-γ in clinical trials yielded controversial results (32–37). After the discovery of Th17 cells and their crucial roles in animal models of various autoimmune diseases including autoimmune arthritis, the roles of IL-17, and Th17 subset have been extensively studied in RA. However, SF of RA contains only low levels of IL-17 and only a small number of Th17 cells (38–41). Moreover, phase III clinical trials using IL-17 inhibitors in RA patients showed no significant benefit over the currently approved biologic agents (42, 43). These clinical results suggest that a single intervention of IL-17 may not be sufficient to ameliorate chronic joint inflammation in RA. Recent studies on the plasticity of Th17 cells in RA and juvenile idiopathic arthritis (JIA) may provide a possible explanation for these observations. That is, a substantial population of IFN-γ^+^ T cells in SF has been reported to co-express CCR6 and CD161, the surface markers of human Th17 cells, and therefore, Th17 cells may be converted to a Th1-like phenotype via an intermediate state of IFN-γ^+^ IL-17^+^ T cells when they encounter an IL-12^high^ environment, which is seen in SF of RA and JIA (44, 45). Such phenotypic changes of Th17 cells in RA joints may account for the failure of the clinical trials using IL-17 inhibitors.

Ex-Th17 cells in RA joints seem to critically contribute to the progress of RA, at least in part by producing GM-CSF (46). The pathogenic role of GM-CSF in RA has been investigated and recent clinical trials using GM-CSF inhibitors have shown significant efficacy (47–50). It is thus likely that pathogenic Th17 cells may have different roles at different phases of RA: IL-17-producing Th17 cells for the initiation in an early phase and GM-CSF-producing ex-Th17 cells for chronic inflammation in a later phase. Following the initiation of arthritis, the majority of Th17 cells become IFN-γ^+^ ex-Th17 cells in response to the IL-12-abundant environment in arthritic joints and begin to actively produce GM-CSF together with other GM-CSF-producing cells such as FLSs and synovial ILCs. A GM-CSF-rich environment in SF results in the activation of synovial macrophages and induction of abundant pro-inflammatory cytokines such as IL-1, IL-6, and TNFα, leading to chronic bone destruction. The plasticity of Th17 cells toward the Th1-like phenotype is rarely observed in SKG arthritis model but can be seen in other murine models of autoimmune disease (25). For example, IL-17 fate mapping mice revealed that highly pathogenic IFN-γ^+^ or GM-CSF^+^ T cells in the spinal cord of EAE mice were exclusively originated from Th17 cells (51). It is hoped that further elucidation of the molecular mechanisms of Th17 plasticity in autoimmune models would help to develop effective means for controlling pathogenic ex-Th17 cells in RA.

The effect of TNFα signaling on pathogenic T cells has been considered stimulatory, but the kinetics of Th17 cells in RA patients after treatment with TNF inhibitors seems to be variable (52). The ratio of Th17 cells to regulatory T (Treg) cells in RA patients was significantly higher than that in healthy controls (53). *In vitro* stimulation with TNFα directly and indirectly facilitated Th17 differentiation, which was consistent with the correlation of the therapeutic efficacy of TNF inhibitors in RA patients with decreased Th17/Treg ratios in the peripheral blood (54–57). However, in RA patients who were non-respondent to treatment with TNF inhibitors, the frequency of Th17 cells in their peripheral blood was paradoxically increased after the treatment (57, 58). The underlying mechanisms of this paradoxical clinical outcome remain unclear, but it could be due to distinct immunological effects of TNFα signaling via TNF receptor 1 (TNFR1) and TNFR2 as well as inflammatory cells expressing different levels of these receptors (52). For example, specific ablation of TNFα signaling via TNFR1, but not TNFR2, upregulated IL-12/IL-23 p40 expression in macrophages driving Th17 responses (59). Similarly, monocytes from non-responder RA patients, who showed increased Th17 responses after treatment with TNF inhibitors, produced more IL-12/IL-23 p40 (58). These findings taken together suggest that the therapeutic effects of TNF inhibitors on chronic joint inflammation are variable in RA patients because of their heterogenous cellular responses via TNFR1 and TNFR2 signaling and variable compositions of synoviocytes and their expression of the two receptors.

## Regulatory T Cells in RA

Treg cells expressing the transcription factor Foxp3 are indispensable for maintaining immunological self-tolerance and suppressing the development of various autoimmune diseases including autoimmune arthritis (60). However, there are few reports on interactions between Treg cells and FLSs, both in human RA and animal models. Treg cells in autoimmune arthritis have been discussed with regard to the instability and impaired function of Treg cells under inflammatory conditions in inflamed joints (61). Among pro-inflammatory cytokines, IL-6 is a well-characterized cytokine that determines the balance of the differentiation of Th17/Treg cells in the periphery (54, 62–64). Indeed, the treatment of active RA with tocilizumab, a humanized anti-IL-6 receptor antibody, significantly reduced clinical disease activity along with a significant decrease of Th17 cells and an increase of Treg cells in frequency in the peripheral blood (65). This indicates that the high levels of systemic IL-6 in RA patients tip the balance toward the dominance of Th17 cells. Abundant IL-6 in SF of RA largely produced by FLSs and synovial macrophages may also influence the differentiation and expansion of bystander Th cells infiltrating arthritic joints.

Immunomodulatory roles of TNFα in the stability and suppressive functions of Treg cells remain controversial. The defective function of Treg cells was initially reported under TNFα-abundant environments as seen in the peripheral blood and arthritic joints of RA, and several possible mechanisms have been proposed. For example, high levels of TNFα may prevent Treg function by inhibiting the formation of the immunological synapse (IS) between Tregs and APCs. The dynamics of Protein kinase C θ (PKC-θ) in IS appear to be reciprocally regulated in effector T and Treg cells: the recruitment of PKC-θ to IS was necessary for full activation of effector T cells whereas the PKC-θ recruitment reduced suppressive activity of Treg cells. Notably, high levels of TNFα enhanced PKC-θ recruitment to IS in Treg cells and inhibited their suppression activity, while PKC-θ blockade protected Treg cells from inactivation by TNFα and restored suppressive function (66). In contrast, disc large homolog 1 (Dlgh1), a scaffolding protein, was recruited to IS by 4-fold more in Treg cells than in effector T cells, which enhanced suppressive function of Treg cells. Treg cells from active RA patients or from healthy controls treated with TNFα showed diminished Dlgh1 recruitment and reduced suppressive function. In addition, silencing of Dlgh1 gene expression abrogated human Treg cell function (67). Furthermore, the suppressive function of Treg cells in the presence of TNFα was modulated by the dephosphorylation at the Ser418 site of FOXP3 through protein phosphatase 1 and in contrast, the treatment with anti-TNFα antibodies restored Treg function in RA patients (68). These findings collectively indicate that chronic inflammation inhibits the suppressive function of Treg cells in RA in part due to high levels of TNFα which modulate FOXP3 phosphorylation as well as regulate the expression and shuttling of PKC-θ and Dlgh1 in a Treg cell-intrinsic manner. However, this concept was recently challenged. According to reports with different experimental conditions, TNFα did not inhibit the suppressive function of human Treg cells, but rather enhanced the expression of CD25 and Foxp3 in the presence of IL-2, and further promoted the proliferation and survival of Treg cells through the direct effect of TNFα on TNFR 2 in a Treg-cell intrinsic manner to control effector T cells (69, 70). The impact of TNFα on effector T cells should also be considered as mentioned above, and effector T subsets in TNF-abundant environments may be more resistant to the suppression by Treg cells at least in part through the costimulatory effects of TNFα on effector T cells. Further investigation is necessary to reconcile this discrepancy.

## Concluding Remark

Biological agents have brought a paradigm shift in RA treatment. However, it still remains elusive how anti-cytokine agents modulate inflammatory properties of T cells, FLSs, and ILCs in the synovial tissue over the course of the treatments, and how the treatments impact interactions between effector immune cells and FLSs in chronic joint inflammation. Animal models such as SKG mice are useful for addressing these issues. Recent studies including our own have indeed revealed the roles of anti-RPL23A autoantibody and GM-CSF commonly found in SKG mice and RA patients. The studies have also uncovered a previously unappreciated aspect of an inflammatory cascade mediated by arthritogenic Th17 cells interacting with hematopoietic and non-hematopoietic cells in the synovial tissue. Further study will facilitate our understanding of the molecular and cellular basis of chronic joint inflammation and help to devise a new RA treatment with better specificity and efficacy.

## Author Contributions

YT, KH, and SS wrote and edited the manuscript.

### Conflict of Interest Statement

The authors declare that the research was conducted in the absence of any commercial or financial relationships that could be construed as a potential conflict of interest.
